# Social inequalities in the use of formal and informal home care in older women: evidence from a large UK cohort study

**DOI:** 10.1093/ageing/afaf279

**Published:** 2025-10-02

**Authors:** Matthew Quinn, Sasha Shepperd, Sarah Floud

**Affiliations:** Nuffield Department of Population Health, University of Oxford, Oxford, UK; Applied Health Research Unit, Nuffield Department of Population Health, University of Oxford, Oxford, UK; Cancer Epidemiology Unit, Nuffield Department of Population Health, University of Oxford, Oxford, UK

**Keywords:** socio-economic inequalities, older people, unpaid carers, social care–home care, formal care

## Abstract

**Background:**

At a time when access to publicly funded social care is constrained relative to need, older people with care needs with the least resources may disproportionately rely on informal care.

**Methods:**

In 2020–21, 66,604 participants from the UK Million Women Study were invited to complete an online survey, which included questions on pre-pandemic receipt of home care. Responses were combined with data collected at recruitment in 1998, a 2010 re-survey, and hospital admissions in 2017–19. We used multivariable logistic regression to assess the association between education level and area deprivation quintile with formal or informal care use, with adjustment for pre-disposing characteristics (age), enabling resources (household composition and size) and care need (including self-rated health and co-morbidities).

**Results:**

A total of 44,523 women completed the survey; 43,756 were eligible for analysis (mean age 75.6). A total of 1407 (3.2%) received informal care, 544 received formal care (1.2%) and 255 (0.6%) received both. Compared to those with university degrees, those with no qualifications were more likely to receive informal care (OR: 1.50, 95% CI: 1.23–1.84) and less likely to receive formal care (OR: 0.39, 95% CI: 0.25–0.60). The most deprived were more likely to receive informal care (OR: 1.34, 95% CI: 1.09–1.65) compared to the least deprived, and there was a trend across quintiles (*P*_trend_ = .02).

**Conclusion:**

This is the largest UK study to assess variation in social care use by education and deprivation. We found inequities in care that may disadvantage older women in deprived areas and with lower levels of education, and their informal carers.

## Key points

- Few studies have explored the relationship between socio-economic status and social care in the home in the UK and elsewhere.
- We identified inequalities in home care use in the UK after adjusting for the major determinants of care use, such as care need.
- Older women with no qualifications were more likely to use informal care than women with university degrees.
- Conversely, older women with no qualifications were less likely to use formal home care than women with university degrees.
- Reforms to social care are needed to equitably meet the care needs of older people and plan for a growing ageing population.

## Introduction

Social care supports independence in old age by providing assistance with daily activities for those with care needs, and is part of the continuum for achieving universal health coverage [1, 2]. In England, it is estimated that 22% of adults aged 65 or over who live at home require support with at least one activity of daily living, rising to 43% of people aged 85 or over [3, 4]. As the population ages and the number of years lived with disability increases, it is inevitable that the demand for social care will continue to rise [5, 6]. However, the accessibility of publicly funded social care is increasingly constrained relative to need; there has been a 5% decline in the proportion of people accessing care funded or provided by local authorities between 2015/16 and 2023/24, despite a 9% increase in support requests [7].

Dual pressures of increasing demand and constrained supply mean that more care is provided by unpaid informal carers, with an estimated 2.1 million older people receiving informal care provided by 5.5 million informal carers in 2021 [8, 9]. By contrast, in England as of 2022/23, 0.58 million adults (1% total population) received publicly funded long-term home care; approximately two-thirds were older adults (aged 65 and over) and one-third adults of working age (aged 18–64) [10]. This reliance on informal care is not cost-free. Half of informal carers in England provide high-intensity care (≥20 h of care each week) [9]. The demands of caring may lead caregivers to reduce their working hours or exit the workforce, and could negatively impact their mental and physical health [11–14]. Whilst informal care may be a preferred option for some, those who cannot access public or privately funded formal home care may be obliged to rely on informal care to meet their care needs.

Assessing the use of home care across the UK is complicated by the different policies on eligibility for public funded care across its constituent nations, and a lack of data due to the mix of public and private care providers. In England and Wales individuals with savings above a certain threshold (£23 250 in England, £50 000 in Wales) have to self-fund their home or residential care [15]. In Scotland and Northern Ireland, personal care at home is free to those assessed as having a care need, although care in Scotland that falls outside of personal or nursing care is means tested with an upper limit of £29 750 [15]. A key feature of all four systems is a care needs assessment to qualify for publicly funded care [16]. Delays in receiving this assessment act as an additional barrier for a significant proportion of those requesting care, with 430 000 individuals waiting for care assessments in England in 2023 [17].

In England, ‘fairness and accessibility’ are key aspects of the government’s vision for social care, but there is limited data to test how well this holds [1, 18, 19]. The few UK studies that have adjusted for care need to assess if those with the same needs are receiving the same care have used data from the English Longitudinal Study of Ageing (ELSA) [18, 19]. These studies, and evidence from other countries, indicate that informal unpaid care is more likely to be used by those who are more deprived, and formal paid care by those who are less deprived [20–23]. The Netherlands is an exception and has relatively high levels of social care spending compared to the UK, with older people who are more deprived more likely to receive formal care [24, 25]. This study aimed to explore whether the use of formal and informal home care in the UK varies by education and deprivation, both indicators of socio-economic status (SES), after accounting for other determinants of social care such as care need.

## Methods

### Study design

The Million Women Study (MWS) is a population-based UK cohort study that recruited 1.3 million participants between 1996 and 2001 from 66 NHS breast screening centres across England and Scotland [26]. Women from the cohort (*N* = 66 604) were invited to complete an online survey between October 2020 and May 2021 on their experiences of the Covid-19 pandemic, including questions on health and use of formal (public or private) and informal (unpaid) home care before and during the COVID-19 pandemic [27]. We supplemented this survey data with data collected at recruitment of the MWS participants in median year 1998 (IQR 1998–99), at re-survey in median year 2010 (IQR 2009–11) and from NHS hospital admissions data during 2017–19 [28, 29]. Ethical approval was provided by the East of England-Cambridge South Research Ethics Committee (REC 97/5/001). All participants gave consent for follow-up through medical records.

Informal or formal care receipt was determined based on responses to the question ‘During 2020, did someone regularly provide help with some of your personal daily activities and domestic tasks (e.g. washing, dressing, cooking) that you could not manage alone’. Respondents then specified the type and timing of care. Those reporting care from a husband/partner, family, friend or neighbour were classified as informal care receivers. Those who received care from a paid professional were classified as receiving formal care. Only individuals receiving care before, or before and during, the COVID-19 outbreak were classified as receiving care in the main analyses (Supplementary Appendix Tables S1–S3). Individuals could receive both informal and formal care.

Education was a three-level categorical variable based on the highest qualification at recruitment: degree-level qualifications (college or university), completion of secondary (A-levels or O-levels) or technical (nursing, teaching, clerical or commercial) qualifications, or no qualifications.

At recruitment, participants were assigned a Townsend score from the 1991 census based on the location of their postcode within small areas (census enumeration district in England, census output area in Scotland) [30]. The Townsend score is a deprivation index calculated using small area-level data on unemployment, non-car ownership, non-home ownership and household overcrowding [30]. Quintiles were based on the relative distribution of the deprivation index within the recruitment cohort.

Potential confounders associated with the use of formal or informal care were grouped according to Andersen’s behavioural model of health services use [31]. These included ‘pre-disposing characteristics’ (age groups: <75, 75–79 and ≥80), ‘enabling resources’ (living with a partner, child or grandchild, and household size: living alone, 1 co-habitant, 2 or more co-habitants) and ‘care need’ (self-rated health status, shielding status, co-morbidities, other social care). Self-rated health status (excellent, good, fair or poor) pre-March 2020 and shielding status (also known as clinically extremely vulnerable, indicating a higher risk condition such as cancer or on treatment causing immunosuppression) were determined from the COVID-19 questionnaire [32, 33]. Information on existing health conditions (cancer, dementia and cardiovascular disease) came from linked hospital admissions data from 01 January 2017 to 31 December 2019 and self-report of diseases on the 2010 re-survey, where available [29] (Supplementary Appendix Table S4). Formal care receipt is an important predictor of informal care need (and vice-versa). However, previous literature suggests that formal and informal care receipt have the potential to be complementary and therefore correlated [34]. We tested for multicollinearity to assess if there was a linear association between the predictor covariates within the ‘care need’ confounder group. The geographic region (Southern England, Midlands, Northern England and Scotland) was considered as an effect-modifier.

### Statistical analysis

Analysis was conducted using Stata 17 [35]. We used logistic regression to examine the association of education or deprivation with the use of formal care or informal care, adjusting for potential confounders, yielding odds ratios (OR) and 95% confidence intervals (95% CI). The impact of adjusting for groups of variables (pre-disposing, enabling resources and care need) to each model was assessed by the percentage change in the log OR.

Likelihood-ratio tests were used to assess trend across deprivation quintiles, heterogeneity across education level and interaction between final models and geographical region, given the different social care policies in England and the devolved nations. The impact of missing values was assessed by imputing missing SES variables under the assumption they belonged to the lowest and highest education levels and the least and most deprived quintiles of deprivation, and examining the effect on the final models. A further sensitivity analysis was performed with formal or informal care removed from the models where they were included as covariates.

## Results

A total of 44 523 participants responded to the COVID-19 questionnaire (67% of those invited). We excluded 24 individuals (0.05%) in care or nursing homes, 336 (0.75%) with missing education and 394 (0.88%) with missing deprivation. A total of 13 participants missing formal (*n* = 9) or informal care (*n* = 4) status were excluded. This left 43 756 participants for the analysis. There were no significant differences in characteristics between participants excluded for missing SES data and those included (Supplementary Appendix Tables S5 and S6). All participants had answered questions on care status, and 41 586 (95.0%) had completed the 2010 re-survey. A total of 2360 (5.4%) had a hospital admission with cancer, 1636 (3.7%) with cardiovascular disease and 46 (0.1%) with dementia.

Participants had a mean age of 75.6 years (SD 3.7). A total of 1707/43 756 (3.9%) received home care; 1407/43 756 (3.2%) received informal care and 544/43 756 formal care (1.2%), of which 255/43 756 (0.6%) received both care types. Informal care was mostly received from unpaid sources inside the home (*n* = 1101/1407, 78.3% of informal care). Formal care was primarily received from paid/professional sources outside of the home (*n* = 519/544, 95.4% of formal care) (Supplementary Appendix Table S3). Informal care receipt increased by 25.2% and formal care receipt decreased by 5.9% during the COVID-19 outbreak (Supplementary Appendix Table S3). Informal and formal care, and education and deprivation, were only weakly correlated (*r* = 0.28; *r =* 0.04, respectively).

Women with no qualifications received more informal care (4.9%) and less formal care (0.9%) than women with degree-level qualifications (2.9% and 1.5% respectively) (Table 1). Women in the most deprived group received more informal care (4.3%) but similar levels of formal care (1.4%) compared to the least deprived (3.0% and 1.3% respectively) (Table 2). Women with no qualifications and from the most deprived quintile reported the highest levels of fair/poor health (26.0% and 22.4% respectively) (Tables 1 and 2).

**Table 1:** Characteristics of participants by education level. A total of 255 participants received both formal and informal care. Values not included where the number of participants <10.

	Education		
Characteristics	No qualifications (*n* = 3024)	Secondary or technical qualifications (*n* = 21 631)	Tertiary qualifications (*n* = 19 101)
**Age**			
Mean (SD)	75.7 (3.7)	75.6 (3.7)	75.5 (3.7)
**Area *n* (%)**			
Southern England	1619 (53.5%)	11 855 (54.8%)	9785 (51.2%)
Midlands	557 (18.4%)	3350 (15.5%)	3098 (16.2%)
Northern England	722 (23.9%)	5316 (24.6%)	4724 (24.7%)
Scotland	126 (4.2%)	1110 (5.1%)	1494 (7.8%)
**Care status *n* (%)**			
No care	2861 (94.6%)	20 813 (96.2%)	18 386 (96.3%)
Informal care	148 (4.9%)	702 (3.2%)	557 (2.9%)
Formal care	26 (0.9%)	232 (1.1%)	286 (1.5%)
**Deprivation quintile *n* (%)**			
Q1 (least deprived)	634 (21.0%)	6220 (28.8%)	5526 (28.9%)
Q2	655 (21.7%)	5361 (24.8%)	4573 (23.9%)
Q3	670 (22.2%)	4598 (21.3%)	4085 (21.4%)
Q4	579 (19.1%)	3609 (16.7%)	3306 (17.3%)
Q5 (most deprived)	486 (16.1%)	1843 (8.5%)	1611 (8.4%)
**Co-habitants *n* (%)**			
None	1001 (33.2%)	6925 (32.1%)	6021 (31.6%)
One	1829 (60.7%)	13 530 (62.7%)	12 103 (63.5%)
Two or more	184 (6.1%)	1130 (5.2%)	949 (5.0%)
Missing	10 (0.3%)	46 (0.2%)	28 (0.1%)
**Partner *n* (%)**			
Lives with partner	1879 (62.1%)	13 769 (63.7%)	12 294 (64.4%)
**Child *n* (%)**			
Lives with child	204 (6.7%)	1331 (6.2%)	1071 (5.6%)
**Grandchild *n* (%)**			
Lives with grandchild	73 (2.4%)	369 (1.7%)	300 (1.6%)
**Health status *n* (%)**			
Fair/Poor	787 (26.0%)	4028 (18.6%)	3243 (17.0%)
**Shielding *n* (%)**			
Yes	392 (13.0%)	2137 (9.9%)	1657 (8.7%)
**History of cancer *n* (%)**			
Yes	502 (16.6%)	3553 (16.4%)	3114 (16.3%)
**History of dementia *n* (%)**			
Yes	<10	34 (0.2%)	27 (0.1%)
**History of cardiovascular disease *n* (%)**			
Yes	294 (9.7%)	1676 (7.7%)	1218 (6.4%)

**Table 2:** Characteristics of participants by deprivation quintile. A total of 255 participants received both formal and informal care. Values not included where the number of participants <10.

	Deprivation quintile (DQ)				
Characteristics	DQ 5 (most deprived) (*n* = 3940)	DQ 4 (*n* = 7494)	DQ 3 (*n* = 9353)	DQ 2 (*n* = 10 589)	DQ 1 (least deprived) (*n* = 12 380)
**Age**					
Mean (SD)	75.3 (3.7)	75.6 (3.7)	75.6 (3.7)	75.7 (3.8)	75.5 (3.7)
**Area *n* (%)**					
Southern England	2033 (51.6%)	4383 (58.5%)	5433 (58.1%)	5846 (55.2%)	5564 (44.9%)
Midlands	567 (14.4%)	1146 (15.3%)	1498 (16.0%)	1662 (15.7%)	2132 (17.2%)
Northern England	1212 (30.8%)	1580 (21.1%)	1917 (20.5%)	2494 (23.6%)	3559 (28.7%)
Scotland	128 (3.2%)	385 (5.1%)	505 (5.4%)	587 (5.5%)	1125 (9.1%)
**Care status *n* (%)**					
No care	3737 (94.8%)	7205 (96.1%)	8993 (96.2%)	10 201 (96.3%)	11 924 (96.3%)
Informal care	169 (4.3%)	237 (3.2%)	301 (3.2%)	327 (3.1%)	373 (3.0%)
Formal care	56 (1.4%)	93 (1.2%)	123 (1.3%)	116 (1.1%)	156 (1.3%)
**Education level *n* (%)**					
No qualifications	1611 (40.9%)	3306 (44.1%)	4085 (43.7%)	4573 (43.2%)	5526 (44.6%)
Secondary/technical	1843 (46.8%)	3609 (48.2%)	4598 (49.2%)	5361 (50.6%)	6220 (50.2%)
Tertiary	486 (12.3%)	579 (7.7%)	670 (7.2%)	655 (6.2%)	634 (5.1%)
**Co-habitants *n* (%)**					
None	1625 (41.3%)	2607 (34.9%)	3017 (32.3%)	3226 (30.5%)	3472 (28.1%)
One	2059 (52.4%)	4454 (59.6%)	5820 (62.4%)	6824 (64.5%)	8305 (67.2%)
Two or more	247 (6.3%)	413 (5.5%)	492 (5.3%)	527 (5.0%)	584 (4.7%)
Missing	<10	20 (0.3%)	24 (0.3%)	12 (0.1%)	19 (0.2%)
**Partner *n* (%)**					
Lives with partner	2056 (52.2%)	4497 (60.0%)	5926 (63.4%)	6959 (65.7%)	8504 (68.7%)
**Child *n* (%)**					
Lives with child	290 (7.4%)	479 (6.4%)	555 (5.9%)	637 (6.0%)	645 (5.2%)
**Grandchild *n* (%)**					
Lives with grandchild	90 (2.3%)	146 (1.9%)	162 (1.7%)	160 (1.5%)	184 (1.5%)
**Health status *n* (%)**					
Fair/Poor	883 (22.4%)	1443 (19.3%)	1708 (18.3%)	1885 (17.8%)	2139 (17.3%)
**Shielding *n* (%)**					
Yes	507 (12.9%)	743 (9.9%)	890 (9.5%)	966 (9.1%)	1080 (8.7%)
**History of cancer *n* (%)**					
Yes	659 (16.7%)	1238 (16.5%)	1543 (16.5%)	1731 (16.3%)	1998 (16.1%)
**History of dementia *n* (%)**					
Yes	<10	11 (0.1%)	22 (0.2%)	<10	21 (0.2%)
**History of cardiovascular disease *n* (%)**					
Yes	342 (8.7%)	573 (7.6%)	671 (7.2%)	730 (6.9%)	872 (7.0%)

Compared to the main MWS study cohort, 6.9% of participants reported having no qualifications vs 43.8% of the MWS cohort at recruitment; 9.0% were in the most deprived quintile and 28.3% in the least deprived quintile (compared to 19.9% and 20.1% of the original MWS cohort, respectively).

**Figure 1:**
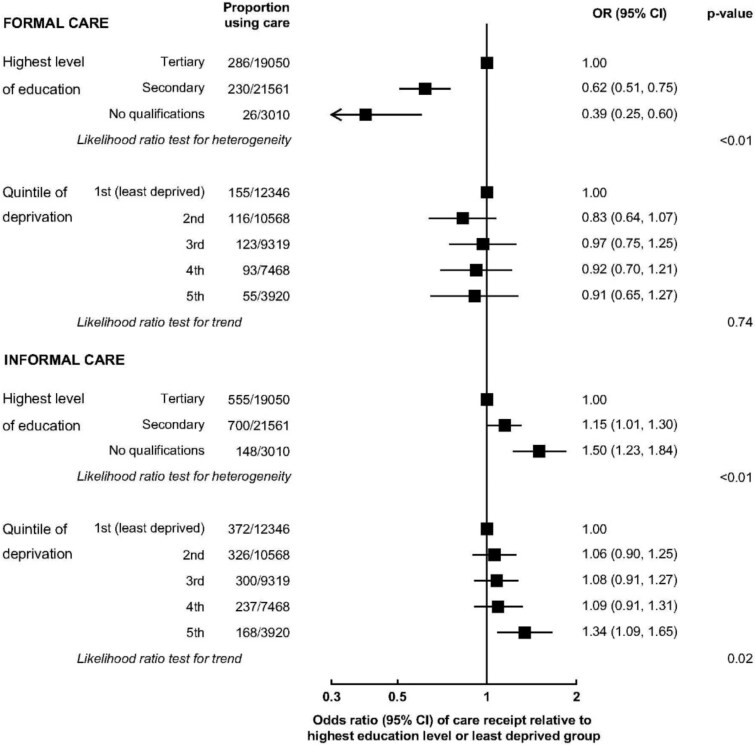
Odds ratios of care use by markers of socioeconomic status. All models were adjusted for pre-disposing characteristics (age), enabling resources (living with partner/child/grandchild, household size) and care need (self-rated health, shielding status, other care [informal or formal] received, history of cancer/dementia/cardiovascular disease).

Figure 1 shows the fully adjusted associations between education and deprivation and the use of formal and informal care. Lower education was associated with lower odds of receiving formal care (*P*_het_ < .01) and higher odds of receiving informal care (*P*_het_ < .01). Individuals with no qualifications had lower odds of formal care use (OR: 0.39, 95% CI: 0.25–0.60) and higher odds of informal care use (OR: 1.50, 1.23–1.84) compared with those with degree-level qualifications. Those with secondary/technical qualifications had lower odds of formal care use (OR: 0.62, 0.51–0.75) and higher odds of informal care (OR: 1.15, 1.01–1.30) compared with those who had degree-level qualifications. The most deprived, compared to the least deprived, were more likely to use informal care (OR: 1.34, 1.09–1.65); but the association with use of formal care was uncertain (OR: 0.91, 0.65–1.27). There was a trend in informal care use across deprivation (*P*_trend_ = .02), but not in formal care (*P*_trend_ = .74).

**Figure 2:**
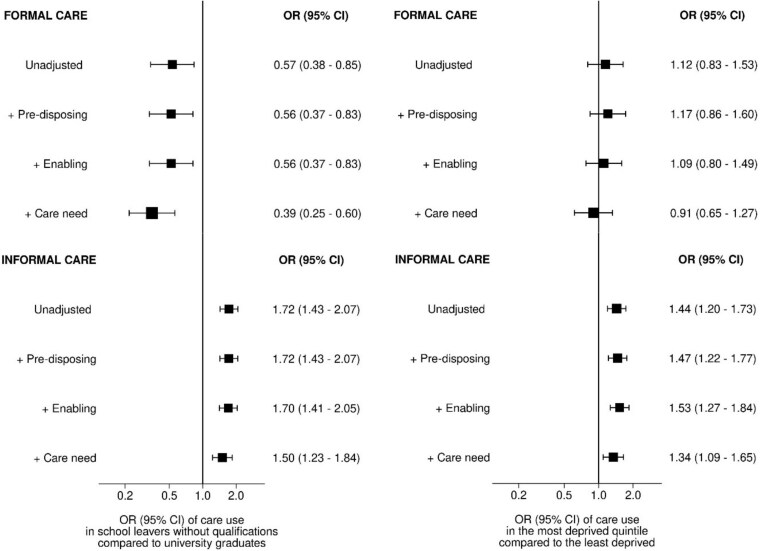
Odds ratios of care use by SES, with sequential adjustment for covariates. Adjustment variables were grouped into pre-disposing characteristics (age), enabling resources (living with partner/child/grandchild, household size) and care need (self-rated health, shielding status, other care [informal or formal] received, history of cancer/dementia/cardiovascular disease).

Figure 2 shows the ORs for informal and formal care for those with no qualifications (compared with degree-level qualifications) and in the most deprived (compared to the least), with sequential adjustments for groups of covariates. The association between education and informal care was attenuated (23% decrease in the log OR) on the addition of care need variables, whereas the association between education and formal care was strengthened on the addition of the care need variables (61% decrease in the log OR). There was an attenuation (30% decrease in the log OR) of the association between deprivation and informal care when care need variables were added, but no significant change in the association between deprivation and formal care.

No significant interaction was found between education or deprivation and geographic region (Supplementary Appendix Table S7). Our results were essentially unchanged in sensitivity analyses when missing SES values were imputed (Supplementary Appendix Tables S8–S11). Removal of formal care as a covariate from the education and informal care model resulted in an attenuation of the relationship between education level and informal care (OR for no qualifications vs tertiary qualifications 1.29, 1.06–1.57). Removal of formal or informal care as covariates from other models made no substantive difference to results (Supplementary Appendix Table S13). There was little evidence of multicollinearity between formal and informal care (variance inflation factor close to 1.0), and therefore the models were adjusted for the presence of formal care (in the informal care model) and informal care (in the formal care model) as markers of care need.

## Discussion

In a large sample of older women in the UK, we found that, despite reporting worse health, older people with no educational qualifications and from the most deprived areas disproportionately relied on informal care. Those with no qualifications were less likely to receive formal care than those with higher levels of education. Similar results were reported by the cross-sectional Survey of Health, Ageing and Retirement in Europe (data collected 2004, 2007 and 2017), and analyses of ELSA data [18–21]. Education is a sensitive marker of lifetime earnings, which may affect the affordability of paid care and drive use of unpaid care among those with lower levels of education [36–38]. Other factors, such as a preference for informal care [39–43] or living close to family may lead to greater informal care use [20, 21].

Our findings contribute to the evidence on inequalities in the receipt of care in the home by older adults in the UK, with two important implications. First is that the current system of means testing and assessment for eligibility for care is failing to provide equitable access to formal home care for older adults [44]. Proposals have been made to address this in England by raising the means test threshold [1]. However, this will not fully reduce inequalities without addressing waiting times for needs assessments and improving the supply of publicly funded care [1, 45–47]. Secondly, there is a risk of widening health inequalities because of the impact of high-intensity unpaid care on mental health and employment, which disproportionately affects people with fewer resources [12–14, 48].

The UK saw a rapid rise in informal care provision over the COVID-19 pandemic, when an additional 4.5 million people became carers [49, 50]. Whilst this number had declined towards pre-pandemic levels by March 2021, the background trend is one of increasing reliance on informal care [51]. In the UK, the number of informal carers is predicted to increase to 9 million by 2037, driven by population ageing and the NHS plan to shift older people’s healthcare into the community [52, 53]. Demographic changes such as a falling birth rate and longer working lives with rising pensionable age may also impact informal care availability and result in a care deficit [54, 55].

A strength of our analysis is the size of the study population and the inclusion of self-reported receipt of publicly and privately funded home care. This is important given the relative lack of accurate data on unpaid care (particularly given under-reporting of informal caring responsibilities among census respondents), and the very limited publicly available data on receipt of privately funded home care and other types of social care [56–59]. Limitations include a lack of data on the proximity of family members living outside the home, though we did adjust for the availability of care from family inside the home (by adjusting for co-residence with partners, children and grandchildren). The use of area-level deprivation at recruitment rather than an individual-level measure may explain the unexpected absence of a relationship between increasing deprivation and less use of formal care, given individuals may have moved from that area or the characteristics of the area may have changed. It is possible that we underestimated inequalities in the use of formal home care, due to a lower proportion of women living in deprivation and with no qualifications, compared with the main MWS cohort. Additionally, over half the sample lived in the south of England (*n* = 23 259, 59%), with a small proportion living in northern England (*n* = 10 762, 25%) and Scotland (*n* = 2730, 6%). Again, this might have underestimated the differences in the use of informal and formal care as there are higher rates of intensive informal care provision in the north of England than the south, and a lower proportion of people who pay for home care [60]. Of note, Scotland provides free home care for older people assessed as needing care [15]. Despite this, we found no significant effect modification by region. Whilst participants reported receipt of paid formal care, it was not possible to determine the source of funding (self-funded or other), how frequently care was received and for how long. Residual confounding is a possibility, as several associations (for example, between education and informal care receipt) were attenuated upon adjustment for care need.

To avoid results that reflected a change in care arrangements due to the COVID-19 pandemic, our analysis was limited to care received prior to, or prior to and during, the pandemic, and we excluded those whose care was limited to the period of the pandemic. While we adjusted for a wide range of variables measuring care need, for example, shielding status during COVID-19 and self-reported health, we were not able to include more direct measures of functional ability (e.g. measures of performance of activities of daily living) that might have improved the measurement of need for care.

This paper builds on previous UK evidence, mainly from ELSA, on inequalities in social care access. Surveying women who participated in the MWS was minimally burdensome for the women involved, and utilised pre-existing socioeconomic and healthcare data to answer questions related to social care. Given the significant gaps in social care research, the use of large population-based health cohorts combined with routinely collected data provides an opportunity for social care research, particularly in the context of caring as an important determinant of health [61].

## Conclusion

This is the largest UK study to assess variation in social care use by education and deprivation. Our findings emphasise the importance of policy that avoids a disproportionate care burden on those with fewer resources to ensure equitable access and use of social care.

## Supplementary Material

Supplementary_materials_afaf279

## Data Availability

Access to the MWS data is through an open-access data application. Full details of the Million Women Study data access policy and application process can be viewed online (https://www.ceu.ox.ac.uk/research/the-million-women-study/data-access-and-sharing).
