# Supplementary material for: Social inequalities in the use of formal and informal home care in older women: evidence from a large UK cohort study
Source: Age Ageing. 2025 Oct 2;54(10):afaf279. doi: 10.1093/ageing/afaf279 (PMC12490715; doi:10.1093/ageing/afaf279)
Supplement: Supplementary_materials_afaf279 [file supplementary_materials_afaf279.docx]

**Social inequalities in the use of formal and informal home care in older women: evidence from a large UK cohort study**

**Supplementary material**

Contents

[Appendix Table S1 3](#_Toc202367535)

[Appendix Table S2 3](#_Toc202367587)

[Appendix Table S3 4](#_Toc202367588)

[Appendix Table S4 5](#_Toc202367589)

[Appendix Table S5 6](#_Toc202367590)

[Appendix Table S6 7](#_Toc202367591)

[Appendix Table S7 8](#_Toc202367592)

[Appendix Table S8 8](#_Toc202367593)

[Appendix Table S9 8](#_Toc202367594)

[Appendix Table S10 8](#_Toc202367595)

[Appendix Table S11 8](#_Toc202367596)

[Appendix Table S12 9](#_Toc202367597)

[Appendix Table S13 10](#_Toc202367598)

| **Who provided this care?** | **Question B: Husband, partner or family member living with me** | | | | | | |
| --- | --- | --- | --- | --- | --- | --- | --- |
| **Question C: Family, friend or neighbour not living with me** |  | **Yes, only before the coronavirus outbreak** | **Yes, both before and during the coronavirus outbreak** | **Yes, only during the coronavirus outbreak** | **Not applicable** | **No** | **Prefer not to answer** |
|  | **Yes only before the coronavirus outbreak** | **Informal care** | **Informal care** | **Informal care** | **Informal care** | **Informal care** | **Informal care** |
|  | **Yes, both before and during the coronavirus outbreak** | **Informal care** | **Informal care** | **Informal care** | **Informal care** | **Informal care** | **Informal care** |
|  | **Yes, only during the coronavirus outbreak** | **Informal care** | **Informal care** | **No informal care** | **No informal care** | **No informal care** | **No informal care** |
|  | **Not applicable** | **Informal care** | **Informal care** | **No informal care** | **No informal care** | **No informal care** | **Missing** |
|  | **No** | **Informal care** | **Informal care** | **No informal care** | **No informal care** | **No informal care** | **Missing** |
|  | **Prefer not to answer** | **Informal care** | **Informal care** | **No informal care** | **Missing** | **Missing** | **Missing** |

Appendix Table S1: Table presenting answer combinations to questions B and C, following question A “During 2020, did someone regularly provide help with some of your personal daily activities and domestic tasks (e.g. washing, dressing, cooking) that you could not manage alone”. “Informal care”, “No informal care” and “Missing” denote how participant was coded based on answer combinations.

| **Who provided this care?** | **Question D: Paid/professional carer living with me** | | | | | | |
| --- | --- | --- | --- | --- | --- | --- | --- |
| **Question E: Paid/professional carer not living with me** |  | **Yes, only before the coronavirus outbreak** | **Yes, both before and during the coronavirus outbreak** | **Yes, only during the coronavirus outbreak** | **No** | **Not applicable** | **Prefer not to answer** |
|  | **Yes only before the coronavirus outbreak** | **Formal care** | **Formal care** | **Formal care** | **Formal care** | **Formal care** | **Formal care** |
|  | **Yes, both before and during the coronavirus outbreak** | **Formal care** | **Formal care** | **Formal care** | **Formal care** | **Formal care** | **Formal care** |
|  | **Yes, only during the coronavirus outbreak** | **Formal care** | **Formal care** | **No formal care** | **No formal care** | **No formal care** | **No formal care** |
|  | **No** | **Formal care** | **Formal care** | **No formal care** | **No formal care** | **No formal care** | **Missing** |
|  | **Not applicable** | **Formal care** | **Formal care** | **No formal care** | **No formal care** | **No formal care** | **Missing** |
|  | **Prefer not to answer** | **Formal care** | **Formal care** | **No formal care** | **Missing** | **Missing** | **Missing** |

Appendix Table S2*:* Table presenting answer combinations to questions D and E, following question A ““During 2020, did someone regularly provide help with some of your personal daily activities and domestic tasks (e.g. washing, dressing, cooking) that you could not manage alone”. “Formal care”, “No formal care” and “Missing” denote how participant was coded based on answer combinations.

|  | *Care from inside home* | *Care from outside of home* | *Care from both* | *Total individuals receiving care* |
| --- | --- | --- | --- | --- |
| Before Covid-19 outbreak | | | |  |
| *Informal care* | 1,101 (78%) | 537 (38%) | 231 (16%) | 1407 |
| *Formal care* | 53 (10%) | 519 (95%) | 28 (5%) | 544 |
| During Covid-19 outbreak | | | |  |
| *Informal care* | 1,311 (74%) | 842 (48%) | 391 (22%) | 1762 |
| *Formal care* | 59 (12%) | 489 (96%) | 36 (7%) | 512 |

Appendix Table S3*:* Summary of data from Appendix Tables S1-2.

| Variable | Category of report |
| --- | --- |
| History of dementia | Hospital diagnosis of dementia (ICD-10 F00-F03, G30, G31) |
|  | Self-reported – Alzheimer’s disease |
|  | Self-reported – Other dementia |
| History of cancer | Hospital diagnosis of cancer (ICD-10 C00–C97) |
|  | Self-reported - Breast cancer |
|  | Self-reported - Cervical cancer/pre-cancer |
|  | Self-reported - Endometrial cancer |
|  | Self-reported - Bowel cancer |
|  | Self-reported - Lung cancer |
|  | Self-reported - Skin cancer |
| History of cardiovascular disease | Hospital diagnosis of cardiovascular disease (ICD-10 I20–I25) |
|  | Self-reported – History of heart attack |
|  | Self-reported – History of stroke |
|  | Self-reported – History of other heart disease |

Appendix Table S4*:* Generation of history of dementia, cancer and cardiovascular disease variables.

| Variables | Eligible  (n = 43,756) | Missing education level  (n = 336) | p-value |
| --- | --- | --- | --- |
| Age |  |  | 0.14 |
| Mean (SD) | 75.6 (3.7) | 75.3 (3.7) |  |
| Area *n* (%) |  |  | 0.97 |
| Southern England | 23,259 (53.2%) | 177 (52.7%) |  |
| Midlands | 7,005 (16.0%) | 57 (17.0%) |  |
| Northern England | 10,762 (24.6%) | 81 (24.1%) |  |
| Scotland | 2,730 (6.2%) | 21 (6.2%) |  |
| Deprivation quintile *n* (%) |  |  | 0.50 |
| Q1 (least deprived) | 12,380 (28.3%) | 90 (27.1%) |  |
| Q2 | 10,589 (24.2%) | 84 (25.3%) |  |
| Q3 | 9,353 (21.4%) | 70 (21.1%) |  |
| Q4 | 7,494 (17.1%) | 50 (15.1%) |  |
| Q5 (most deprived) | 3,940 (9.0%) | 38 (11.4%) |  |
| Co-habitants *n* (%) |  |  | 0.46 |
| None | 13,947 (31.9%) | 98 (29.2%) |  |
| One | 27,462 (62.9%) | 224 (66.7%) |  |
| Two or more | 2,263 (5.2%) | 14 (4.2%) |  |
| Partner *n* (%) |  |  | 0.40 |
| Lives with partner | 27,942 (63.9%) | 222 (66.1%) |  |
| Child *n* (%) |  |  | 0.36 |
| Lives with child | 2,606 (6.0%) | 24 (7.1%) |  |
| Grandchild *n* (%) |  |  | 0.47 |
| Lives with grandchild | 742 (1.7%) | <10 |  |
| Health status *n* (%) |  |  | 0.79 |
| Excellent/Good | 8,058 (18.4%) | 60 (17.9%) |  |
| Shielding *n* (%) |  |  | 0.99 |
| Yes | 4,186 (9.6%) | 32 (9.6%) |  |
| History of cancer *n* (%) |  |  | 0.38 |
| Yes | 7,169 (16.4%) | 61 (18.2%) |  |
| History of dementia *n* (%) |  |  | 0.47 |
| Yes | 68 (0.2%) | <10 |  |
| History of cardiovascular disease *n* (%) |  |  | 0.47 |
| Yes | 3,188 (7.3%) | 21 (6.2%) |  |
| Formal care |  |  | 0.56 |
| Yes | 544 (1.2%) | <10 |  |
| Informal care |  |  | 0.20 |
| Yes | 1,407 (3.2%) | 15 (4.5%) |  |

Appendix Table S5: Characteristics of participants by missing education level compared with those eligible. For the continuous variable (age), p-values were obtained using a two-sample t-test. For categorical variables, p-values were obtained using Pearson’s chi-squared test of association.

| Variables | Eligible  (n = 43,756) | Missing deprivation quintile  (n = 394) | p-value |
| --- | --- | --- | --- |
| Age |  |  | 0.003 |
| Mean (SD) | 75.6 (3.7) | 76.1 (4.1) |  |
| Area *n* (%) |  |  | <0.001 |
| Southern England | 23,259 (53.2%) | 252 (64.0%) |  |
| Midlands | 7,005 (16.0%) | 36 (9.1%) |  |
| Northern England | 10,762 (24.6%) | 96 (24.4%) |  |
| Scotland | 2,730 (6.2%) | 10 (2.5%) |  |
| Education level *n* (%) |  |  | 0.79 |
| Tertiary qualifications | 19,101 (43.7%) | 168 (42.6%) |  |
| Secondary/technical qualifications | 21,631 (49.4%) | 201 (51.0%) |  |
| No qualifications | 3,024 (6.9%) | 25 (6.3%) |  |
| Co-habitants *n* (%) |  |  | 0.24 |
| None | 13,947 (31.9%) | 134 (34.0%) |  |
| One | 27,462 (62.9%) | 239 (60.7%) |  |
| Two or more | 2,263 (5.2%) | 21 (5.3%) |  |
| Partner *n* (%) |  |  | 0.19 |
| Lives with partner | 27,942 (63.9%) | 239 (60.7%) |  |
| Child *n* (%) |  |  | 0.34 |
| Lives with child | 2,606 (6.0%) | 19 (4.8%) |  |
| Grandchild *n* (%) |  |  | 0.20 |
| Lives with grandchild | 742 (1.7%) | 10 (2.5%) |  |
| Health status *n* (%) |  |  | 0.94 |
| Excellent/Good | 8,058 (18.4%) | 72 (18.3%) |  |
| Shielding *n* (%) |  |  | 0.66 |
| Yes | 4,186 (9.6%) | 35 (8.9%) |  |
| History of cancer *n* (%) |  |  | 0.55 |
| Yes | 7,169 (16.4%) | 69 (17.5%) |  |
| History of dementia *n* (%) |  |  | 0.62 |
| Yes | 68 (0.2%) | <10 |  |
| History of cardiovascular disease *n* (%) |  |  | 0.95 |
| Yes | 3,188 (7.3%) | 29 (7.4%) |  |
| Formal care |  |  | 0.34 |
| Yes | 544 (1.2%) | <10 |  |
| Informal care |  |  | 0.63 |
| Yes | 1,407 (3.2%) | 11 (2.8%) |  |

Appendix Table S6: Characteristics of participants by missing deprivation quintile compared with those eligible. For the continuous variable (age), p-values were obtained using a two-sample t-test. For categorical variables, p-values were obtained using Pearson’s chi-squared test of association.

|  | **Informal care** | **Formal care** |
| --- | --- | --- |
| **Education** | X^2^_(6)_ 6.14_,_ p = 0.41 | X^2^_(6)_ 1.66 p = 0.95 |
| **Deprivation** | X^2^_(12)_ 11.54, p = 0.48 | X^2^_(12)_ 8.13, p = 0.78 |
| Appendix Table S7: Effect modification by geographic region, models compared with and without interaction term with geographic region variable. | | |

|  | Informal care  OR (95% CI) | Formal care  OR (95% CI) |
| --- | --- | --- |
| No qualifications | 1.57 (1.30 to 1.91) | 0.38 (0.25 to 0.57) |
| Secondary/technical qualifications | 1.15 (1.02 to 1.31) | 0.61 (0.51 to 0.74) |
| Tertiary qualifications | 1.00 | 1.00 |

Appendix Table S8*:* All missing exposure variables imputed as no qualifications.

|  | Informal care  OR (95% CI) | Formal care  OR (95% CI) |
| --- | --- | --- |
| DQ 5 (most deprived) | 1.32 (1.08 to 1.61) | 0.92 (0.67 to 1.27) |
| DQ 1 (least deprived) | 1.00 | 1.00 |

Appendix Table S9: All missing exposure variables imputed as most deprived quintile.

|  | Informal care  OR (95% CI) | Formal care  OR (95% CI) |
| --- | --- | --- |
| No qualifications | 1.52 (1.24 to 1.86) | 0.38 (0.25 to 0.58) |
| Secondary/technical qualifications | 1.14 (1.01 to 1.29) | 0.62 (0.52 to 0.75) |
| Tertiary qualifications | 1.00 | 1.00 |

Appendix Table S10: All missing exposure variables imputed as tertiary qualifications.

|  | Informal care | Formal care |
| --- | --- | --- |
| DQ 5 (most deprived) | 1.36 (1.11 to 1.66) | 0.89 (0.62 to 1.24) |
| DQ 1 (least deprived) | 1.00 | 1.00 |

Appendix Table S11*:* All missing exposure variables imputed as least deprived.

| Variable | Source |
| --- | --- |
| Care receipt (Formal & Informal Care) | Covid-19 Questionnaire (2020-21) |
| Education level | Recruitment (1997) |
| Quintile of deprivation | Recruitment (1997) |
| Age group | Recruitment (1997) |
| Living with a partner/child/grandchild | Covid-19 Questionnaire (2020-21) |
| Household size | Covid-19 Questionnaire (2020-21) |
| Overall self-rated health status | Covid-19 Questionnaire (2020-21) |
| Shielding status during the Covid-19 pandemic | Covid-19 Questionnaire (2020-21) |
| Geographic region | Recruitment (1997) |
| History of dementia | HES (2017-19) & Re-survey (2011) |
| History of cancer | HES (2017-19) & Re-survey (2011) |
| History of cardiovascular disease | HES (2017-19) & Re-survey (2011) |

Appendix Table S12*:* Source of model variables.

| **Informal care-education model (formal care removed as co-variate)** | | | |
| --- | --- | --- | --- |
| **Education level** | **OR** | **LCI** | **UCI** |
| Tertiary qualifications | 1.00 | - | - |
| Secondary/Technical qualifications | 1.06 | 0.94 | 1.19 |
| No qualifications | 1.29 | 1.06 | 1.57 |
| **Informal care-deprivation model (formal care removed as co-variate)** | | | |
| **Deprivation quintile** | **OR** | **LCI** | **UCI** |
| DQ1 | 1.0 | - | - |
| DQ2 | 1.03 | 0.88 | 1.21 |
| DQ3 | 1.06 | 0.90 | 1.25 |
| DQ4 | 1.05 | 0.89 | 1.25 |
| DQ5 | 1.30 | 1.06 | 1.58 |
| **Formal care-education model (informal care removed as co-variate)** | | | |
| **Education level** | **OR** | **LCI** | **UCI** |
| Tertiary qualifications | 1.00 | - | - |
| Secondary/Technical qualifications | 0.67 | 0.56 | 0.79 |
| No qualifications | 0.45 | 0.30 | 0.68 |
| **Formal care-deprivation model (informal care removed as co-variate)** | | | |
| **Deprivation quintile** | **OR** | **LCI** | **UCI** |
| DQ1 | 1.0 | - | - |
| DQ2 | 0.86 | 0.67 | 1.09 |
| DQ3 | 1.01 | 0.79 | 1.28 |
| DQ4 | 0.93 | 0.71 | 1.21 |
| DQ5 | 0.95 | 0.70 | 1.31 |

Appendix Table S13*:* Non-outcome care status removed as co-variate.
